# The Swedish medical birth register during five decades: documentation of the content and quality of the register

**DOI:** 10.1007/s10654-022-00947-5

**Published:** 2023-01-03

**Authors:** Sven Cnattingius, Karin Källén, Anna Sandström, Henny Rydberg, Helena Månsson, Olof Stephansson, Thomas Frisell, Jonas F. Ludvigsson

**Affiliations:** 1 Clinical Epidemiology Division, Department of Medicine, Karolinska Institutet, Solna, Sweden; 2 Department of Clinical Sciences, Centre of Reproduction Epidemiology, Tornblad Institute, Lund University, Lund, Sweden; 3 Department of Women’s Health, Division of Obstetrics, Karolinska University Hospital, Stockholm, Sweden; 4 Statistics Unit 1, Department of Registers and Statistics, National Board of Health and Welfare, Stockholm, Sweden; 5 Department of Medical Epidemiology and Biostatistics, Karolinska Institutet, 17177 Stockholm, Sweden; 6 Department of Pediatrics, Örebro University Hospital, Örebro, Sweden; 7 Department of Medicine, Columbia University College of Physicians and Surgeons, New York, NY USA

**Keywords:** Antenatal care, Birth infant, Nationwide, Newborn, Population-based pregnancy, Register, Sweden

## Abstract

Pregnancy-related factors are important for short- and long-term health in mothers and offspring. The nationwide population-based Swedish Medical Birth Register (MBR) was established in 1973. The present study describes the content and quality of the MBR, using original MBR data, Swedish-language and international publications based on the MBR.

The MBR includes around 98% of all births in Sweden. From 1982 onwards, the MBR is based on prospectively recorded information in standardized antenatal, obstetric, and neonatal records. When the mother and infant are discharged from hospital, this information is forwarded to the MBR, which is updated annually. Maternal data include information from first antenatal visit on self-reported obstetric history, infertility, diseases, medication use, cohabitation status, smoking and snuff use, self-reported height and measured weight, allowing calculation of body mass index. Birth and neonatal data include date and time of birth, mode of delivery, singleton or multiple birth, gestational age, stillbirth, birth weight, birth length, head circumference, infant sex, Apgar scores, and maternal and infant diagnoses/procedures, including neonatal care. The overall quality of the MBR is very high, owing to the semi-automated data extraction from the standardized regional electronic health records, Sweden’s universal access to antenatal care, and the possibility to compare mothers and offspring to the Total Population Register in order to identify missing records. Through the unique personal identity numbers of mothers and live-born offspring, the MBR can be linked to other health registers. The Swedish MBR contains high-quality pregnancy-related information on more than 5 million births during five decades.

**Supplementary Information:**

The online version contains supplementary material available at 10.1007/s10654-022-00947-5.

## Introduction

Since its inception in 1973, the nation-wide Swedish Medical Birth Register (MBR) has served as a key source of information for clinical and epidemiological studies of maternal and perinatal characteristics, as well as short and long-term health in mothers and offspring.

Part of the great value of the MBR for research stems from the personal identity numbers (PINs) for mothers and live-born infants, a person-unique number [1], which allows deterministic linkage to other national Swedish registers [2–6], specialized health care quality registers [7], and research specific data collections. Researchers can request individual level data from the MBR and other registers for research and statistics purposes, given that required ethical approval has been obtained [8]. Such data are usually pseudonymized, i.e., the mother’s and infant’s PINs are replaced by other unique numbers, which cannot be used for linkage outside the specific research project. Certain aggregated data can be accessed free of charge also by non-researchers, through a dedicated webpage from the National Board of Health and Welfare (NBHW) [9], and it is possible to order tailored aggregated statistics. Despite its great value for research, there is little contemporary documentation of the contents and quality of the MBR available in English [10, 11].

### Data collection

Swedish antenatal and obstetric care is publicly funded, almost all pregnant women attend antenatal care regularly [12, 13], and give birth in hospitals [14]. All healthcare providers in Sweden are obliged by law to report antenatal, obstetric, and neonatal data to the NBHW, where the records are merged, quality checked, and annually released for use as the MBR (Law concerning registers of health data: 1998:543, and regulation specifically about the MBR: 2001:708). From 1973 to 1982, information was manually extracted from antenatal, obstetric, and neonatal records, and transferred to a paper form, which was sent to the NBHW, where information was computerized. From 1982 onwards, the MBR has been based on copies of the standardized medical antenatal, obstetric, and neonatal records which, after completion, were transferred to the MBR. Manual data entering of information from these records to MBR was successively replaced by scanning, which resulted in improved register quality. These records have undergone minor revisions, but have remained unchanged since 1998. Electronic reporting began in 2007, and in 2021 only 2 of 21 regions submitted paper records, which were then computerized at the NBHW. The electronic reports consist of extracts from each healthcare region’s electronic health records system, which closely resemble the earlier paper records.

Recording of data included in the MBR starts at the mother’s first visit to antenatal care (usually at 8 to 10 gestational weeks), and ends when the mother and infant are discharged from hospital. Data is hence prospectively recorded in antenatal, obstetric, and neonatal care. Pregnancy data are usually recorded by midwives working in outpatient antenatal care, while obstetric and neonatal data are recorded during and after delivery by midwives and physicians.

### Variables included in the MBR

The MBR currently includes information on more than 200 variables (Supplementary Table  1). The majority of variables are directly transferred from the standardized clinical records, where the information is generally collected through pre-specified check-boxes or through assigned diagnostic or procedure codes. Some variables in the MBR are transferred from other registers or calculated at the NBHW (sources of information are provided in Supplementary Table 1).

At the first antenatal visit, most pregnant women spend around one hour with the midwife for a structured interview and an examination. Date of the first antenatal visit is recorded, which later enables calculation of gestational age at registration to antenatal care. Mother’s date of birth (the first six digits in mother’s PIN) is noted, which later enables calculation of the mother’s age at childbirth. From this first visit, the MBR records, in check-boxes, self-reported information about previous obstetric history (number of livebirths, stillbirths, miscarriages, and ectopic pregnancies), presence/absence for a pre-defined list of diseases (listed in Supplementary Table 1), cohabitation status, current and previous smoking and snuff use. Self-reported information about maternal height (in cm) is recorded, while weight (in kg) is measured with the pregnant woman wearing light indoor clothes. Date of last menstrual period (LMP) and estimated date of delivery by LMP are recorded by the midwife. Since 1982, the woman is also asked if she and her partner had difficulties in conceiving, and if so, how long they actively tried before succeeding (time-to-pregnancy) [15]. Women who answer yes are further asked if they received any fertility assistance and if so, what kind.

During pregnancy, self-reported information about medication use is recorded in free text in the antenatal record, and this information is later translated into ATC-codes. The medication use includes both over-the-counter (OTC) and prescription medications. Estimated date of delivery by ultrasound is recorded. Starting in the 1990s, all pregnant women in Sweden are offered an ultrasound examination around 18 gestational weeks, and about 95% or more accept this offer [16]. Number of antenatal visits are recorded, and smoking and snuff use is also recorded in gestational week 30–32.

Onset of delivery is recorded as spontaneous, induced, or caesarean section (CS) before start of labour contractions. During and at delivery, information about use and type of pain-relieving analgesics and anesthesia are recorded. Birth and neonatal data include delivery clinic, date and time of birth, mode of delivery (vaginal non-instrumental, vaginal instrumental [vacuum extraction and forceps are noted separately], and CS), maternal lacerations, singleton or multiple birth, stillbirth, birth weight, birth length, head circumference, and sex of infant. Gestational age at delivery is coded in weeks and days according to an algorithm where gestational age estimated by ultrasound is preferred when available, followed by gestational age estimated by date of LMP, and (least preferred) a note in clinical records at birth. Apgar scores are noted at 1, 5, and 10 min. Maternal and infant diagnoses and operations/procedures are coded, using the Swedish versions of International Classification of Diseases (ICD) and Classification of operations and major procedures, respectively [17]. Similar to the National Patient Register(5), ICD-8 was used up until 1986; ICD-9 between 1987 and 1996 (including 1997 in the Skåne region), and ICD-10 since 1997 (since 1998 in the Skåne region). Information on neonatal care, including neonatal intensive care, is also noted at discharge from hospital. Most birth information is recorded by the midwife in charge, but the physician records data on diagnoses and procedures. Of note, the exact dates of maternal and infant diagnoses are not recorded. To these data are added vital data from the government agency Statistics Sweden on residence and mothers’ country of birth. In neonatal deaths (live-born infants who die during their first 27 days of life), information on age of death in days is collected from the Cause of Death Register (held by the NBHW).

The MBR is updated annually, with production usually taking up to 1–2 years. Data on maternal medication use is added after this, due to the extensive work translating free text on medications into relevant ATC codes. The MBR is a “living register” and is corrected over time if inaccuracies are found, but the magnitude of such late revisions is very limited.

### Overall quality and quality assurance of data

The overall quality of the MBR is very high, owing to the semi-automated process through which the data is extracted from regional electronic health records. In addition, number of live births and unique PINs recorded in MBR and the Total Population Register (held by Statistics Sweden) are routinely compared, [4] to identify births missing in the MBR. To increase the accuracy of reported data, each electronic report to the NBHW generates feedback to the rapporteur, including requests for data verifications and completions when improbable combinations of variables, unexpectedly high missingness, or other potential errors are detected.

### Coverage and completeness

The MBR is intended to cover information on all livebirths (irrespective of gestational week) in Sweden as well as stillbirths from 22 completed gestational weeks (from 1973 through June 2008, only stillbirths from 28 gestational weeks were included). Thus, induced abortions and miscarriages prior to 22 gestational weeks are not part of the MBR. Births to non-resident foreign women taking place in Sweden are included, but births by Swedish women taking place outside Sweden are not.

**Fig. 1:**
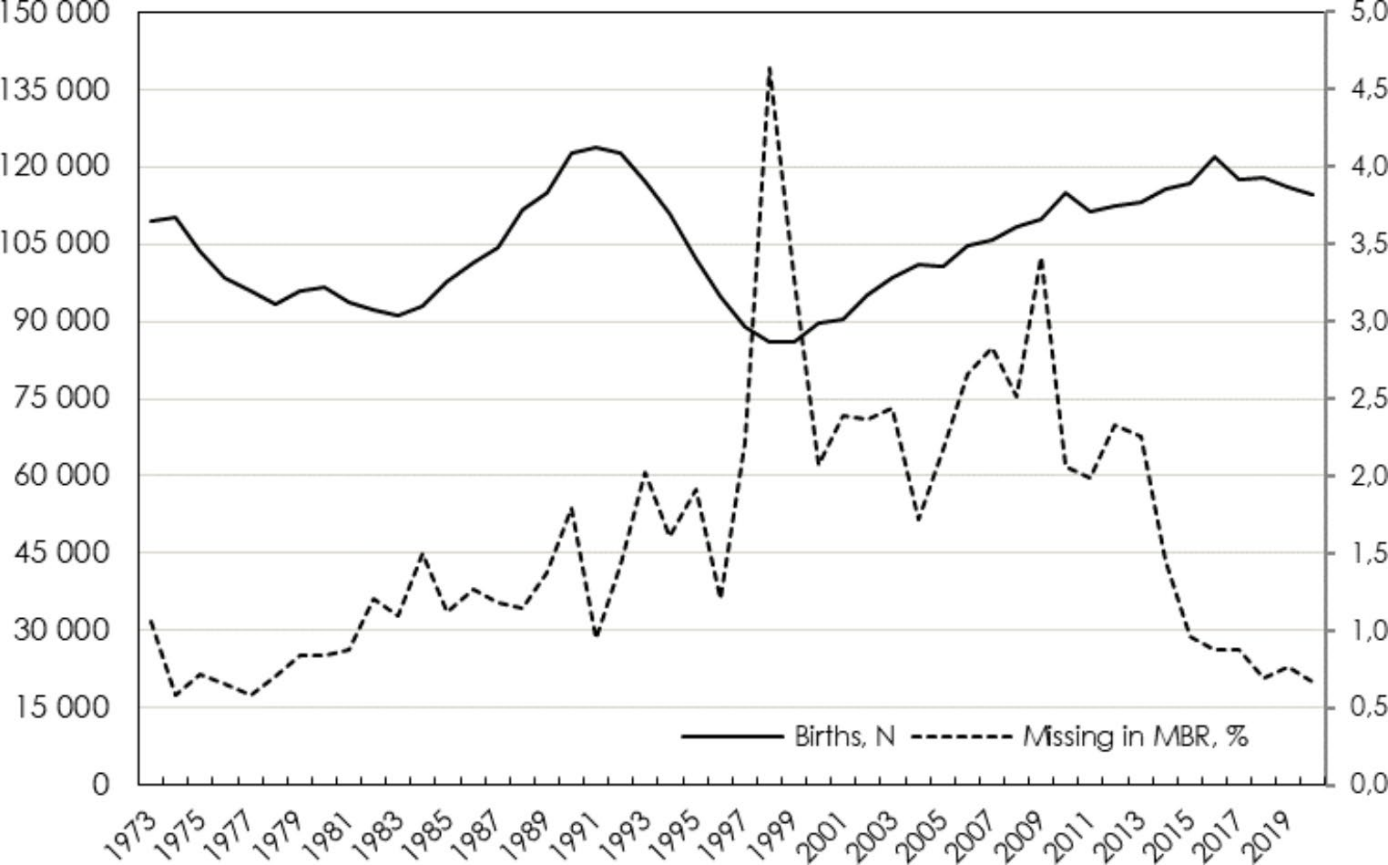
Number of births and national coverage in the Swedish Medical Birth Register (MBR), 1973–2020. Note: Coverage is estimated as the proportion of live births in the Total Population Register, assigned personal identity numbers, that lack a record in the MBR. Some differences are expected: until 2013, children born abroad to Swedish parents (at least a Swedish mother) were recorded in the Total Population Register but not in the MBR. Since 2014, children born abroad to Swedish parents only enter the Total Population Register if either of the parents is a diplomat or working abroad on behalf of the Swedish state(4)

The completeness of the register is assessed by linking births to the PINs of newborns recorded in the Total Population Register [4]. As displayed in Fig. 1, the MBR includes 97–99% of all births in Sweden since 2000, and > 99% since 2015. The historically lowest coverage (95%) was seen in 1998, when one hospital failed to report a large share of its births. Since the MBR also records births of non-resident women in Sweden, the number of births in the MBR currently exceeds that of the Total Population Register [4], which does not include non-residents. Except for historical lapses in reporting from specific hospitals, mothers who are non-residents at time of birth are sometimes temporarily assigned non-valid PINs, which impede the linking between registers. Planned or unplanned home births are rare in Sweden (estimated < 1%), [14] and although possible to report to the register, data on such births may be missing to a high degree.

Overall, missingness is lower for obstetric and neonatal data than for antenatal data. Reduced rates of missingness of not only antenatal data (like smoking, snuff use, maternal height and weight) but also obstetric data (onset of labour and mode of delivery) coincides in time with increasing use of electronic reporting to the MBR, starting in 2007 [18]. Several key variables, including birthweight and gestational length, have been specifically targeted by the quality assurance process and missingness is minimal (< 1%). Many variables (including medical diagnoses, obstetric history, operations and procedures in mothers and infants) are however either based on check-boxes or ICD/procedure codes, making it impossible to distinguish absence of these conditions from missing data.

### Measurement errors

Since reporting shifted to automated extractions from medical records/charts in 1982, MBR data have a high correlation with medical chart data. Measurement errors may of course still occur due to incorrect entering into the patient chart or incorrect coding of diseases or medical/surgical procedures. Pregnant women may e.g., tend to overestimate maternal height, may not want to acknowledge that they are smokers or snuff users, or may not correctly report pre-pregnancy diseases, medication use, and previous obstetric history. From 1999, data are entered through Microsoft Access software, which has decreased error frequency. In 2010, the NBHW retrospectively reviewed a large amount of data and highly unlikely values were corrected or deleted. Currently, logical tests are run to identify inconsistent or unlikely data, but these are not always corrected by reporting units.

In Tables 1 and 2, the validity of commonly used maternal, birth, and neonatal variables have been scored. This validity scoring is based on the joint experience by the authors, after accounting for data collection, data entering and logical check-ups (see Supplementary Table 1 for an example).

**Table 1:** Completeness and quality of commonly used maternal factors

Variables	Variable definition	Missingness by decade	Validity
**Characteristics**			
Age at delivery (completed years)	Date of infant birth - Date of mother’s birth	1990’s 0.1% 2000’s 0.2% 2010’s 0.6%	Excellent, derived from PIN and birth date
Number of infants^a^	Includes present infant	1990’s 0.1% 2000’s 0.2% 2010’s 0.1%	Excellent, calculated from recorded births
Number of deliveries^a^	Includes present delivery	1990’s 0.1% 2000’s 0.2% 2010’s 0.1%	Excellent, calculated from recorded births
Smoking^b^	0 = missing; 1 = non-smoker; 2 = 1–9 cig./day: 3 ≥ 10 cig./day	1990’s 5.7% 2000’s 5.9% 2010’s 5.3%	Very good
Height^c^	In cm	1990’s 17.9% 2000’s 6.7% 2010’s 4.1%	Very good
Weight^d^	In kg	1990-91 100% 1992–1999 14.7% 2000’s 10.7% 2010’s 5.6%	Very good
Country of birth	In free text	1990’s 1.3% 2000’s 1.3% 2010’s 2.2%	Excellent. This information is, for research purposes usually accessible at an aggregated level
**Maternal diagnoses**	By ICD codes 1973-87: ICD-8; 1987-96: ICD-9; 1997-present: ICD-10	Not possible to estimate	
*Common maternal diagnoses*			
Pregestational diabetes^e^	ICD-9: 250; 648 A ICD-10: O240-O243	-“-	Good
Gestational diabetes^e^	ICD-9: 648 W ICD-10: O244	-“-	Good
Primary (essential) hypertension	ICD-9: 401-5; 642 C ICD-10: O10-11; I10-15 (for increased sensitivity, relevant ATC codes from the Prescribed Drug Register can also be used, e.g. C09A-CO9D plus C09X)	-“-	High specificity but lower sensitivity
Gestational hypertension^f^	ICD-9: 642 D and 642X ICD-10: O13	-“-	Good/Very good
Preeclampsia/ Eclampsia^f^	ICD-9: 642E-G ICD-10: O14, O15	-“-	Good
Placental abruption^g^	ICD-9: 641 C ICD-10: O45	-“-	Good

ICD, International Classification of Disease. MBR, Medical Birth Register. PIN, personal identity number. ^a^If the first birth is a twin birth, the variable of number of infants is 2, and number of deliveries variable value is 1. ^b^Self-reported smoking at registration to antenatal care has repeatedly been validated, using cotinine markers (in serum, urine or saliva) [58]. ^c^Values from 100 to 220 cm are accepted. The correlation of maternal height values in successive pregnancies is 0.98 [59]. To reduce missing numbers and measurement error, median height across pregnancies can be calculated. ^d^Usually measured in light indoor clothes at registration to antenatal care. Values from 30 to 200 kg are accepted. By censoring records with unacceptable height and/or weight values, essentially no women (0.007%) with information on maternal height and weight, have BMI values < 12 or > 70. ^e^The prevalences of type 1 and type 2 diabetes in mothers (ICD-10 codes O240 and O241, respectively) were, in a Swedish study of liveborn singleton infants without congenital malformation 0.44% and 0.05%, respectively. Mothers with type 2 diabetes were generally older and more often overweight or obese, compared with mothers with type 1 diabetes. For gestational diabetes, Sweden has a national screening programme, as part of the antenatal care programme) [60]. ^f^The diagnostic ICD-9 codes for gestational hypertension and preeclampsia were compared with individual antenatal and obstetrical charts, using the accepted diagnostic criteria for the conditions (as reported in reference [61]). Among 115 pregnancies coded as gestational hypertension and 148 pregnancies coded as preeclampsia, 97 and 137 pregnancies had gestational hypertension and preeclampsia, respectively, according to the notes in the individual records. Thus, the positive predictive values were 84 and 93%, respectively [62]. For preeclampsia, good validity for register information using ICD-10 codes have been shown in a Danish study [63]. ^g^The prevalence of placental abruption in the MBR (≈ 0.5%) is similar to that in epidemiological studies relying on clinical diagnoses from individual records [44]

**Table 2:** Quality of commonly used birth and neonatal factors

Variable	Variable definition	Missingness by decade	Validity
**Variables used to estimate gestational age** ^**a**^			
-Date of birth	Year-month-date	None	Excellent
-Estimated date by ultrasound	Year-month-date	1990’s 22.5% 2000’s 12.7% 2010’s 6.9%	Excellent
-First day of last menstrual period (LMP)	Year-month-date	1990’s 5.8% 2000’s 8.7% 2010’s 7.7%	Very good
-Estimated date of delivery by LMP	Year-month-date	1990’s 12.1% 2000’s 10.1% 2010’s 9.9%	Very good
-Postnatal assessment of gestational age in weeks	Completed weeks	1990’s 2.3% 2000’s 0.6% 2010’s 0%	Information on underlying method is missing
-Postnatal assessment of gestational age in days in addition to weeks (range 0–6)	Completed days	1990’s 0.5% 2000’s 1.6% 2010’s 0.4%	-“-
**Other birth and neonatal factors**			
Birth weight^b^	In grams	1990’s 0.3% 2000’s 0.3% 2010’s 0.1%	Very good
Sex of infant	Check-box 1 = boy; 2 = girl	None	Excellent
Single or multiple birth	Check-box 1 = single; 2 = multiple	None	Excellent
Stillbirth^c^	Check box recorded at birth	Cannot be calculated	Excellent
Neonatal mortality^d^	In completed (range 0–27) days	Cannot be calculated	Excellent
Apgar scores at 5 minutes^e^	Check-box. Values from 0 to 10 are accepted.	1990’s 1.4% 2000’s 0.4% 2010’s 0.6%	See footnote
Infant diagnoses^f^	ICD codes (ICD-10 since 1997)	Cannot be calculated	See footnote
Infant surgery and procedures^g^	Swedish Classification of Operations and Major Procedures	Cannot be calculated	See footnote

^a^Gestational age in the MBR can be calculated by combining information from date of birth and estimated date of birth by ultrasound, the last menstrual period (LMP), or a postnatal assessment of gestational age in weeks and days. The NBHW has also constructed an algorithm, which, in principle is based on the following hierarchy: gestational age by (a) ultrasound; (b) LMP; (c) as noted in the neonatal record. ^b^For live births, birth weights < 270 and > 6999 g are coded as missing by the MBR. For stillbirths, all values are kept. ^c^From July 2008, stillbirths from 22 completed weeks are included (from 1973 through June 2008, only stillbirths from 28 completed weeks were included). ^d^All births recorded in the MBR are individually matched to the Total Population Register, kept at Statistics Sweden (including information on live births and date of death). Neonatal deaths are recorded in the Cause of Death Register, which includes information of all deaths in Sweden. ^e^Apgar score is also recorded at 1 and 10 min (see Table S1 in web supplement). A cautious approach (to enhance validity) is that, in infants with recorded very low Apgar scores at 5 or 10 min (i.e. “0” or “1”), also check information on other Apgar score values, infant diagnoses, and care at the neonatal ward. Some infants with a recorded Apgar score value at 10 min = 0 (i.e. no sign of life), had full Apgar score (i.e., 10) at 5 min. This likely error was noted during some years, but no such discrepancies in Apgar score values were noted for those with Apgar scores at 1 or 5 min = 0. Reasons for missing information on Apgar score at 10 min could be full Apgar score at 5 min (i.e., additional reporting at 10 min seems to be of little clinical importance). ^f^Validity of infant diagnoses in the MBR should be similar to corresponding information in the Patient Register(5). ^g^In a study of moderately preterm infants, infant surgery and procedure codes were underreported in the MBR, compared with similar information from the Swedish Neonatal Quality Register (see thesis by Altman [64])

The completeness and quality of maternal factors commonly used for research (i.e., age, parity, smoking habits, weight, height, and mother’s country of birth) range from good to excellent (Table 1). The validity of the diagnoses of the most common maternal complications during pregnancy is assessed to be good, although underreporting may exist.

Variables reflecting onset and mode of delivery are primarily based on check-boxes, as noted in the obstetric record (Supplementary Table 1). By using combinations, it is for example possible to define CS before onset of labour and CS during labour. Together with the data collection procedure, the results in Supplementary Table 2 support that the quality of these variables should be assessed as very good.

**Fig. 2:**
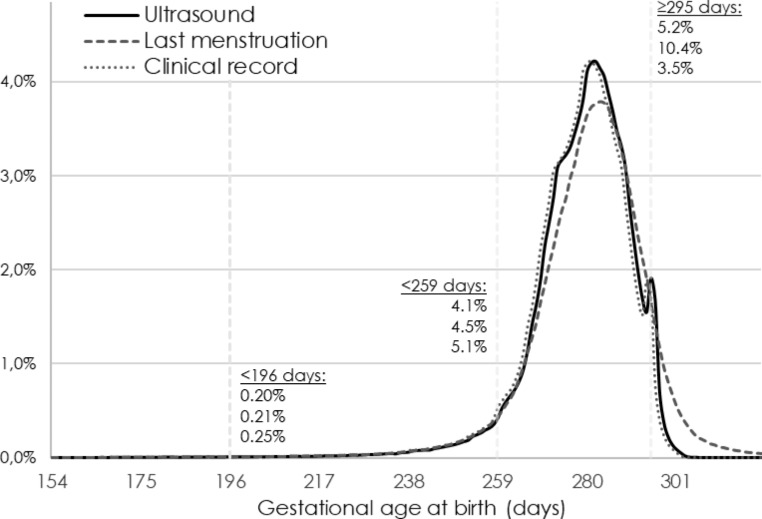
Method for estimating gestational age, all liveborn singleton births, 2000–2020. Note: The distribution in gestational age at birth by method of estimation. Dating from ultrasound and last menstrual period (LMP) have assumed a gestational length of 280 days. Dating by time of LMP overestimates the proportion with longer gestation. The date recorded in clinical records is in recent years based on ultrasound dating, but assumes a gestation of 279 day in some electronic health record systems. Before introduction of electronic health records, or if it not available, there is no underlying information about methods used to estimate gestational age in the clinical records

**Fig. 3:**
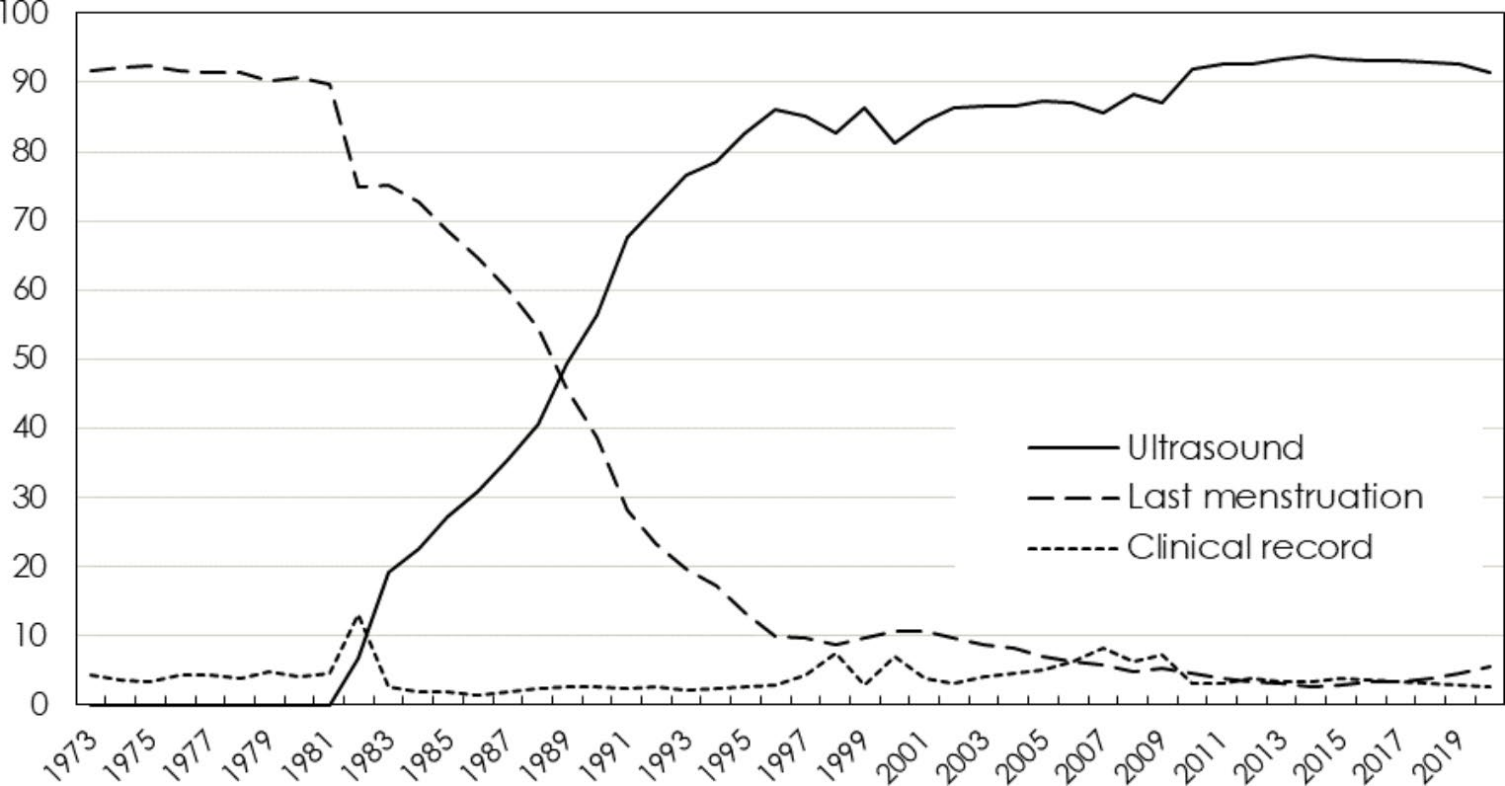
Method for GRDBS (best estimate) gestational age, 1973–2020. Note: The variable for best estimate gestational age (GRDBS) is based on a hierarchical algorithm preferring (1) ultrasound if available, (2) date of last menstrual period (LMP), and (3) clinical record. The algorithm also includes internal comparisons to decide if a date seems congruent with other information. Since the late 1990’s, ultrasound dating dominates as the method eventually used by GRDBS (Best estimate of the duration of pregnancy in days, hierarchical variable)

Correct information on gestational age at birth is of central importance. Expected gestational age (i.e., the assumed duration of a “standard” pregnancy) has varied between 279 and 282 days during different time-periods and in different Swedish counties. Until 2008, the expected gestational duration was generally calculated as one day longer during leap years, when the pregnancy covered February 29th [19]. Gestational age is estimated as the distance in days between the observed date of birth and the date 280 days before the expected date of birth, based on either ultrasound or the first day of the LMP. There is also a recorded estimate of gestational length in the clinical (obstetrical) record. Figure 2 displays the distribution in gestational age at birth if estimated from these different parameters. Estimating gestational age by LMP gives a more symmetric distribution, but with a substantially higher proportion of post-term deliveries (≥ 42 completed gestational weeks). The estimates in the clinical records was previously based on “a clinical estimate”, meaning that the underlying method to estimate gestational age was not reported. During the last two decades, gestational age information is primarily drawn from the ultrasound examination also for the “clinical estimate”, but some electronic health record systems automatically assigns an average expected gestational length of 279 days, which explains why this curve so closely mirrors that based on ultrasound. An algorithm of a “best estimate” gestational age variable is created at the NBHW, which hierarchically prefers (a) the date expected from ultrasound; (b) the LMP; and (c) gestational length as estimated in clinical records. The algorithm only accepts gestational lengths between 154 and 321 days, and makes several internal comparisons between dates, e.g. to dismiss ultrasound dating if too divergent from other information. Since the late 1990s, the “best estimate” has been dominated by ultrasound dating, but in the 1970s it was primarily based on the estimate from the LMP (Fig. 3).

Birth weight, which is reported in grams, and measured immediately after delivery, is another key variable. The MBR accepts values from 270 to 6999 g in live births and all values in stillbirths. By combining information on birth weight and gestational age, it is possible to reveal possible mismatches between birth weight and gestational age. Using the sex-specific Swedish reference curve for normal fetal growth [20], Supplementary Table 3 displays the distribution of birth weight for gestational age in standard deviations (SD). Among 2.2 million liveborn singletons, only 1076 (0.05%) either had a birth weight or gestational age exceeding 5 SD above the average, and only 198 (0.01%) had a value lower than 5 SD below the average. Since these extreme values are unlikely to be correct, it is recommended that they are actively set to missing before use in research.

The completeness and quality of gestational age, birth weight, and other commonly used infant measures are shown in Table 2. Variables reflecting gestational age, birth weight, sex, and mortality are assessed as having very good or excellent quality. It is recommended that very low Apgar score values at 10 min (0–1) should be investigated in more detail (using other information). Norman et al. have examined the validity of infant diagnoses 2012-16 in the MBR/Patient Register and in the Swedish Neonatal Quality Register (SNQ) [21].

A known error relates to multiple births. For same-sex twins born the same day, a twin may be incorrectly matched to his/her sibling’s medical record data, including Apgar score, diagnoses and birthweight etc. This error may have occurred in up to 50% of all same-sex twins (by random, half of the matchings were correct). Since 2020, the NBHW requires the healthcare regions to confirm that the matching of same-sex twins is correct, and a variable (”BPNRQ_FB”, see Supplementary Table 1) has been created to identify PINs that might be incorrectly matched.

### Medical research using the MBR

A PubMed search on Aug 2, 2022 resulted in 927 hits (“medical birth reg*” AND (Sweden or Swedish)). A similar search in the US National Library of Medicine (PMC) yielded 2096 hits. Given that the MBR started in 1973, long-term follow-up of mothers and children are possible (using information from the National Patient [5] and Cause of Death [3] Registers), as well as studies of births in successive generations. By linkage to population-based registers held by Statistics Sweden, birth outcomes can be compared among socio-economic groups (“income” or “highest level of completed education”) and migrants (“mother’s country of birth” and “date of immigration”). Data on emigrations from the Total Population Register [4] will also help calculate a correct follow-up. Linking the MBR and the Multigeneration Register (part of the Total Population Register) enables identification of first-degree relatives other than the mother-offspring dyad; thus, we can compare mothers who are full siblings and also identify the paternal contribution to outcomes in offspring. The MBR has also been used to study specific neonatal, chronic disease and treatment-associated exposures and outcomes by linkages to different National Quality Registers, as the Swedish Neonatal Quality Register (SNQ), the National Quality Register for Assisted Reproduction (Q-IVF) and the Swedish Rheumatology Quality Register (SRQ) [19, 22, 23].

A large number of studies have focused on exposures of public health importance, such as maternal smoking and body-mass index (BMI) [24, 25], and obstetric, neonatal and long-term risks in offspring. By linking successive pregnancies, it has been demonstrated that perinatal risks generally decrease when the mother stops smoking, and increase when the mother gains weight [26, 27]. By identifying full siblings (using data on fathers from the Multigeneration Register), the positive associations between maternal smoking and offspring risks of psychiatric morbidity (e.g. ADHD) found in cohort studies, have not been supported by results from studies using a co-sibling design [28]. This indicates that findings from cohort studies may be due to confounding by genetic or environmental factors shared by siblings [29]. Medication use during pregnancy as well as assisted reproductive techniques have been investigated in large studies, by using similar data from the Nordic countries (Denmark, Norway, Finland, Iceland, and Sweden) [30–34].

Gestational age at birth influences offspring long-term morbidity [35–43] and mortality risks, and risks increase with decreasing gestational age. Women with pregnancy-related complications (notably gestational hypertension, preeclampsia, gestational diabetes, and placental abruption) are at increased risks for morbidity and/or mortality later in life [44–47]. Also other pregnancy-related factors, such as parity and mode of delivery [48], have been linked to future health and comorbidity [49]. While it is well-known that maternal disease may influence pregnancy outcomes [50–52], recent data also indicate that it may have a long-term effect on the health in offspring [53–55].

Researchers apply for data through a two-step process. First, the Swedish Ethical Review Authority must approve the researchers’ study plan [8]. The National Board of Health and Welfare must then evaluate if data can be released under the special conditions set out in the Public Access to Information and Secrecy Act (OSL 2009:400), which include an assessment of whether the requested data are necessary for the proposed research, and of the risk for harming research subjects through release of their personal data. Researchers will be charged for the work incurred on the NBHW in the processing of the data order. Data will be pseudonymized before sent to the researcher.

## Discussion

The nation-wide Swedish MBR started in 1973, and covers more than 5 million births. The MBR has been the basis of more than 1000 research publications. As reviewed in this paper, the prospective data collection, using standardized antenatal, obstetric and neonatal forms, have resulted in high validity of data and near complete national coverage.

Part of the register’s success stems from the Swedish PIN and the country’s system of national registers, and that Swedish healthcare is tax-funded [12], yielding high use of antenatal care and enabling almost all pregnant women and all births in Sweden to be recorded in the MBR. These factors are shared by the other Nordic countries, where similar birth registers are in operation.

Preceded by Norway (1967), the Swedish and the Danish MBRs were founded in the same year (1973) [33]. The Icelandic MBR started in 1981, while Finland initiated its equivalent in 1987. High validity was reported for most diagnoses and procedures in the Danish MBR, although validity was lower for pain relief procedures and uterine rupture (cited through Laugesen et al. [33]). The Danish MBR has, similar to the Swedish MBR, replaced paper forms with electronic reporting. This direct computerized extraction of variables from medical records should ensure similar data quality in the MBRs and medical records.

The large number of pregnancies and births in the Swedish MBR leads to great statistical power, allowing for important subanalyses and exploration of rare but serious exposures and outcomes (such as placental abruption and stillbirth). The population-based design together with the high coverage minimize the risk of selection bias. The MBR contains data which are not present in other national healthcare registers, including smoking, snuff use and body mass index, allowing researchers to examine the role of modifiable risk factors for adverse pregnancy outcomes. Of note, the prevalence of daily smoking in early pregnancy in Sweden has declined from 31% to 1983 to 3.7% in 2020.

The register also has some limitations. Most likely, the MBR has low sensitivity for some infant diagnoses and procedures. The MBR also has a limited ability to capture malformations, since many malformations are diagnosed after the infant is discharged from hospital. In order to increase sensitivity for malformations, some researchers have combined data from the MBR with Patient Register data [56, 57]. There is also a degree of missingness. For example, using information about maternal weight and height in early pregnancy and maternal weight before delivery, it is possible to calculate weight gain during pregnancy. However, from 2010 onwards, this information is missing in 62% of pregnancies. Pregnancies not leading to delivery are not recorded, and there are large regional variations in missingness of tobacco data (i.e. smoking and snuff use) in late pregnancy.

With such a long-standing register, the benefit of comparability over time must be weighed against the need for modernization in contents and production. Ongoing development work at the NBHW aims to improve the production time of the register, hoping to achieve more frequent updates with shorter lag-time in the near future. In conclusion, the Swedish MBR contains pregnancy-related high-quality information on more than 5 million pregnancies and births. These data have been the foundation of extensive research on the mother-offspring dyad, examining both short-term, long-term and cross-generational effects on health.

## Electronic supplementary material

Below is the link to the electronic supplementary material.

Supplementary Material 1

Supplementary Material 2

## Abbreviations

ATC: Anatomical Therapeutic Chemical
CS: Cesarean section
LMP: Last menstrual period
MBR: Medical Birth Register
NBHW: National Board of Health and Welfare
PIN: Personal Identity Number.
