# Supplementary material for: The Swedish medical birth register during five decades: documentation of the content and quality of the register
Source: Eur J Epidemiol. 2023 Jan 3;38(1):109–20. doi: 10.1007/s10654-022-00947-5 (PMC9867659; doi:10.1007/s10654-022-00947-5)
Supplement: Supplementary file 1 — Supplementary Material 1 [file 10654_2022_947_MOESM1_ESM.docx]

**SUPPLEMENT**

**The Swedish Medical Birth Register during five decades:** **documentation of the content and quality of the register**

Sven Cnattingius, Karin Källén, Anna Sandström, Henny Rydberg, Helena Månsson, Olof Stephansson, Thomas Frisell^,^ and Jonas F. Ludvigsson

Page 2: **Supplementary Table 1.** Variables included in the Swedish Medical Birth Register (MBR)

Page 11: **Supplementary Table 2.** Onset of labour and mode of delivery. Swedish Medical Birth Register 2000-2020

Page 12: **Supplementary Table 3.** Distribution of birth weight for gestational age in the Swedish Medical Birth Register (MBR). Live singleton births 2000-2020 (n=2,203,837)

Page 13: **References**

**Supplementary Table 1.** Variables included in the Swedish Medical Birth Register (MBR)

| **Variables** | **Name in MBR** | **Source** | **Comments** |
| --- | --- | --- | --- |
| **Maternal** |  |  |  |
| Personal Identity Number (PIN) | MPNR | Antenatal record |  |
| Date of Birth, text | MFODDAT | Calculated by the NBHW (based on year-month-date in PIN |  |
| Date of birth, numeric | MFODDATN | -“- |  |
| PIN, quality | MPNRQ | Calculated by NBHW |  |
| Place of residence at delivery, 1973-2015 | LKF | Statistics Sweden | County, municipality, parish |
| Place of residence at delivery, 1973 onwards | LK | Statistics Sweden | County, municipality |
| Date of first antenatal visit | INDATMHV | Antenatal record |  |
| Antenatal care clinic (ANC) code | MHVNR | -“- |  |
| Date of first day of last menstrual period (LMP) | SMDAT | Antenatal record | Self-reported at first antenatal visit |
| Estimated date of delivery by LMP | BPSMDAT | -“- |  |
| Estimated date of delivery by ultrasound | BPULDAT | -“- | Ultrasonic examination no later than at 18 gestational weeks |
| Date for stopping taking oral contraceptives | PSTOPDAT | -“- | Self-reported at first antenatal visit |
| Date for extraction of intrauterine contraceptive device (IUD) | SPUTDAT | -“- | -“- |
| Pregnancy with IUD | SPGRAV | Antenatal record - checkbox |  |
| Weight at registration to ANC (measured) | MVIKT | Antenatal record |  |
| Height at registration to ANC | MLANGD | -“- | -“- |
| Smoking and snuff use |  |  |  |
| Smoking 3 months before pregnancy | ROK0 | Antenatal record, check-box | -“- |
| Smoking at registration to ANC | ROK1 | -“- | -“- |
| Smoking at 30-32 gestational weeks | ROK2 | -“- | Self-reported at 30-32 gestational weeks |
| Snuff use 3 months before pregnancy | SNUS0 | -“- | Self-reported at first antenatal visit |
| Snuff use at registration to ANC | SNUS1 | -“- | -“- |
| Snuff use at 30-32 gestational weeks | SNUS2 | -“- | Self-reported at 30-32 gestational weeks |
| Cohabitation status | FAMSIT | -“- | Self-reported at first antenatal visit. Cohabitation status is defined as: living with “the father-to-be”, living alone, or other family composition. Cohabitation status can be combined with the variable reflecting civil status (included 1973-1981, see below) |
| Employment outside the home | ARBETE | -“- | Coded as full-time, part-time, or no employment |
| Type of occupation | YRKE | Antenatal record, free text |  |
| Infertility |  |  | Self-reported at first antenatal visit |
| Infertility, years | OFRIBARN | Antenatal record |  |
| Infertility, no treatment | OFRIATG | Antenatal record, check-box |  |
| Infertility, in vitro fertilization (IVF) | OFRIABF | -“- |  |
| Infertility, stimulation of ovulation | OFRISTIM | -“- |  |
| Infertility, surgery | OFRIKIRU | -“- |  |
| Infertility, intracytoplasmic sperm injection (ICSI) | OFRIICSI | -“- |  |
| Infertility, other treatment | OFRIANN | -“- |  |
| Previous pregnancies |  |  | Self-reported at first antenatal visit |
| Miscarriages, number | TIDSPOAB | Antenatal record, check-box |  |
| Ectopic pregnancies, number | TIDXGRAV | -“- |  |
| Stillbirths, number | TIDDODF | -“- |  |
| Live births, number | TIDLEVF | -“- |  |
| Early neonatal deaths, number | TID7DOD | -“- | Previous death of infant at 0-6 completed days of life |
| Other infant or child deaths, number | TIDSDOD | -“- | Other infant or child deaths (from 7 days and onward) |
| **Maternal diseases** |  |  | Self-reported at first antenatal visit (from 1990 onwards) |
| Recurrent urinary tract infections | URINVINF | -“- |  |
| Chronic renal disease | NJURSJUK | -“- |  |
| Diabetes mellitus (pregestational) | DIABETES | -“- | In 1990-93, this variable may also include previous gestational diabetes |
| Epilepsy | EPILEPSI | -“- |  |
| Asthma | ASTMA | -“- |  |
| Crohn’s disease or ulcerative colitis | ULCOLIT | -“- |  |
| Systemic lupus erythematosus | SLE | -“- |  |
| Primary (essential) hypertension | HYPERTON | -“- |  |
| Number of antenatal visits | BESOK | Calculated by the NBHW |  |
| Local record | LOKREG | Antenatal record | A field enabling local data entry (if wanted) as free text |
| Prenatal diagnostics |  | -“- | The variables of prenatal diagnostics are likely underreported |
| Chorionic villus sample/biopsy (CVB) | CVB | Antenatal record, check-box |  |
| Amniocentesis | AMNIO | -“- |  |
| Date of CVB | CVBDAT | Antenatal record |  |
| Date of amniocentesis | AMNIODAT | -“- |  |
| Annotation of results from amniocentesis | AMNIOANM | -“- |  |
| **Labour and Delivery** |  |  |  |
| Delivery ward - admission | INDATFV | Obstetrical record |  |
| Maternal weight at delivery ward - admission | MVIKTFV | -“- |  |
| Previous caesarean section(s) (CS) | TSECTIO | -“- |  |
| Year(s) for previous CS | TSECAR | -“- |  |
| Spontaneous onset of labour | FLSPONT | Obstetrical record, check-box |  |
| Induction of labour | FLINDUKT | -“- |  |
| CS before onset of labour | SECFORE | -“- |  |
| Anaesthesia during labour: |  | Obstetrical record,  -“- |  |
| epidural | EPIBL |  |  |
| spinal | SPINAL |  |  |
| paracervical blockade | PARABL |  |  |
| pudendal blockade | PUDBL |  |  |
| general anaesthesia | NARKOS |  |  |
| petidine | PETIDIN |  |  |
| infiltration anaesthesia | INFILT |  |  |
| tranquilizers/ sedatives | SEDATIVA |  |  |
| nitrous oxygen | LUSTGAS |  |  |
| acupuncture | AKUPUNKT |  |  |
| water immersion | BAD |  |  |
| sterile water injections | KVADDLAR |  |  |
| transcutaneous nervous stimulations | TNS |  |  |
| hypnosis | HYPNOS |  |  |
| other anaesthetic methods | ANNANSML |  |  |
| no anaesthesia | IFSML |  |  |
| Maternal lacerations: |  | Obstetrical record, check-box | Information from these check-boxes can be complemented with information from maternal diagnoses (ICD-codes). |
|  | CLITORIS |  |  |
|  | VAGINA |  |  |
|  | PERINEUM |  |  |
|  | SFINKTER |  |  |
|  | REKTUM |  |  |
|  | CERVIX |  |  |
| Method of delivery |  |  |  |
| Elective or emergency CS | ELAKUT | Obstetrical record, check-box | Quality assessed as poor. This information can be retrieved from other variables (see Table S2) |
| Vaginal non-instrumental delivery | VAGINAL | -“- |  |
| Vacuum extraction (VE) delivery | SUGKLOCK | -“- | Delivery ended with vacuum extraction |
| Forceps delivery | TANG | -“- | Delivery ended with forceps |
| CS delivery | SECAVSL | -“- |  |
| Forceps used any time during delivery | TANGMARK | Calculated by the NBHW | Information is based on check-boxes, diagnoses, and surgery codes. For method of delivery, this forceps variable, and the corresponding variables for VE and CS are recommended to be used. |
| VE used any time during delivery | SUGMARK | -“- |  |
| Any CS | SECMARK | -“- |  |
| Other surgery at delivery (i.e., besides CS, forceps, and VE) | FLOP | Obstetrical record, surgery codes |  |
| ICD version used for diagnoses | ICD | Calculated by the NBHW | In MBR and other health care registers in Sweden: ICD-8:1983-86; ICD-9 1987-96; ICD-10 1997-present |
| Maternal diagnoses during pregnancy, delivery, and post-partum | MDIAG1 to MDIAG12 | Obstetrical record | Since 1999, there are 12 fields for recording of maternal diagnoses |
| All maternal diagnoses in one text string | MDIAGNOS | -“- |  |
| Maternal surgery or procedures at delivery | FLOP1 to FLOP12 | -“- | Since 1999, there are 12 fields for recording of maternal procedures and operations |
| All maternal surgery or procedures in one text string | MFLOP | -“- |  |
| Date of discharge from hospital - mother | MUTDAT | -“- |  |
| Mother discharged to home or to other care facility | MUTSATT | Obstetrical record, check-box | Noted as: 1=home, 2=to other care |
| Hospital code as reported to NBHW | SJUKHUS | -“- |  |
| Hospital code - corrected | SJUKHUS_S | Calculated by the NBHW (to reduce erroneous hospital codes) | From 2018 onwards, a specific code is also provided for home births |
| Hospital department (ob/gyn far most common) | KLINIK | Obstetrical record |  |
|  |  |  |  |
| **Infant** |  |  |  |
| Personal Identity Number (PIN) | BPNR | Statistics Sweden |  |
| Birth year, infant | AR | Calculated by the NBHW |  |
| Date of Birth, text | BFODDAT | Neonatal record |  |
| Date of birth, numeric | BFODDATN | -“- |  |
| PIN, quality | BPNRQ | Calculated by the NBHW |  |
| Quality of PIN, multiple births | BPNRQ_FB | -“- |  |
| Time of birth (hour, minutes) | FODKL | Neonatal record |  |
| Singleton or multiple birth | BORDF2 | -“- |  |
| In multiple births: birth order and number of births | BORDNRF2 | -“- | For example, if you are a first born triplet, birth order=1 and number of births=3 |
| In multiple births: number of chorioamniotic membranes | HINNANT | -”- |  |
| Presentation at birth | BJUDNING |  | From 1999: 1=occiput anterior; 4:occiput posterior; 6=breech or foot; 0=other. Maternal and infant diagnoses may also be used to define presentation at birth. |
| Stillbirth | DODFOD | -“- | 1=antepartal, 2=intrapartal |
| Time of death in live births (hour, minutes) | DODKL | -“- |  |
| Sex of infant (1=boy, 2=girl) | KON | -“- |  |
| Gestational age – completed weeks | GRVFV | -“- | Underlying method to estimate gestational age is not noted. Still, these estimates (recorded in the clinical record), are, since year 2000 primarily drawn from the ultrasound examination. |
| Gestational age – additional days (0-6) | GRDFV | -“- | -“- |
| Gestational age in weeks - best estimate (hierarchy) | GRVBS | Calculated by the NBHW | Based on an algorithm, which, in principle is based on the following hierarchy: gestational age by a) ultrasound; b) LMP; c) as noted in the neonatal record |
| Best estimate of the duration of pregnancy (gestational age) in days, hierarchical variable | GRDBS | -“- | -“- |
| Method to estimate best estimate of pregnancy duration (gestational age) in each pregnancy | GRMETOD | -“- |  |
| Birth weight, grams | BVIKT | Neonatal record | For live births, values from 270 to 6999 grams are accepted. For stillbirths, all values are accepted |
| Birth length, cm | BLANGDF2 | -“- | Values from 20 to 79 cm are accepted |
| Head circumference (pediatric), cm | HOMF | -“- | Values from 15 to 45 cm are accepted |
| Apgar scores at 1, 5, and 10 minutes  (range 0-10) | APGAR1, APGAR5, and APGAR10 | -“- |  |
| Specified neonatal procedures |  |  |  |
| Ventilation by mask, minutes | VENT | Neonatal record, check-box |  |
| Ventilation by intubation, minutes | INTUB | -“- |  |
| Chest compression (as part of cardiopulmonary resuscitation) minutes | HJMASS | -“- |  |
| Correction of metabolic acidosis | ACIDOSIS | -“- |  |
| Vitamin K (intramuscular or orally) | KVITAMIN | -“- |  |
| Infant diagnoses | BDIAG1 to BDIAG12 | Neonatal record |  |
| All infant diagnoses in one text string | BDIAGNOS | -“- |  |
| Infant surgery or procedures | BFLOP1 to BFLOP12 | -“- |  |
| All infant surgery or procedures in one text string | BFLOP | -“- |  |
| Healthy infant, examined postpartum | FBARN | Neonatal record, check-box | The most commonly reported i “diagnostic” infant information |
| Date of discharge from hospital – infant | BUTDAT | Neonatal record |  |
| Infant stay at hospital >28 days | BUTSATT | -“- | This definition of the variable is used since 1999 |
| Autopsy of infant (1=yes; 2=no) | OBDUKT | Neonatal record, check-box |  |
| Maternal citizenship (free text)^a^ | MNAT | From Statistics Sweden |  |
| Paternal citizenship (free text)^a^ | FNAT | -“- |  |
| Mother’s country of birth (free text)^a^ | MFODLAND | -“- |  |
| Birth order of infant | PARITET | Calculated by the NBHW | Calculation is based on information on number of previous births of each mother (from Statistics Sweden and the MBR) |
| Number of deliveries of each mother | PARITET_F | -“- | Calculation is based on information on number of previous deliveries of each mother (from Statistics Sweden and the MBR) |
| Maternal age (completed years) at delivery | MALDER | -“- | Calculation of maternal age in completed years is based on the mother’s birth date (included in the first 6 digits in mother’s PIN) and infant’s birth date |
| Neonatal mortality in days (0-27 days) | DDAGAR | -“- | Calculation is based on date of death (from Causes of Death Register) and date of birth (from MBR) |
| Fetal/neonatal survival | DKLASS | -“- | Calculation is based on the stillbirth variable in MBR (“DODFOD”), date of death in the Causes of Death Register, and date of birth in MBR: 1=stillbirth; 2=early neonatal mortality (0-6 days); 3=late neonatal mortality (7-27 days) |
| Any congenital malformation | MISSB | -“- | Any diagnosis of congenital malformation (ICD-8 and ICD-9 codes 740-759, and ICD-10 codes Q00-Q99). For studies of environmental exposures, congenital malformation codes that are strictly chromosomal are usually excluded. |
| Small for gestational age^b^ | MSGA | -“- |  |
| Large for gestational age^b^ | MLGA | -“- |  |
| **Medication use** |  |  |  |
| Medication use reported in early or late pregnancy (ATC code), included from 2013. | ATC | Antenatal record, free text | Self-reported at first antenatal visit or in late pregnancy (specified by variable BLANKETT). Includes both prescription and over-the-counter medication. Free text is translated into ATC codes at the NBHW. |
| Medical record from which the medication information originates (MHV1 for early pregnancy or MHV2 for late pregnancy), included from 2013. | BLANKETT | Antenatal record |  |
| Grouping of products that cannot be classified into the ATC system, for example omega-3-products or unspecified vitamins, included from 2013. | GRUPP | Antenatal record, free text | Free text is categorized into predefined groups at the NBHW |
| **Variables previously included in the MBR** |  |  |  |
| Civil status (unmarried, married, divorced), included 1973-81 | CIVIL | Antenatal record | Can be combined with variable “family situation” (see above) |
| Pregnancy diagnoses during pregnancy, four diagnostic fields, included 1973-89 | GDIAG1 to GDIAG4 | -“- |  |
| Penthrane use for analgesia during delivery, included 1982-89 | PENTHRAN | Obstetrical record, check-box |  |
| Placental weight, grams, included 1982-89 | PLACENTA | Obstetrical record |  |
| Elective or non-elective CS, 1=elective; 2=non-elective, included 1982-89 | SECTIO | Obstetrical record |  |
| Medication use reported in early pregnancy, ATC code, included 1995-2012. | ATCM1_01 to ATCM1_17 | Antenatal record, free text | Self-reported at first antenatal visit. Includes both prescription and over-the-counter medication. Free text is translated into ATC codes at the NBHW. Products that cannot be classified into the ATC system are categorized into groups such as 'HEMOFER' or 'OMEGA'. |
| Medication use reported in late pregnancy, ATC code, included 1995-2004.  Includes medication use reported after the first antenatal visit, throughout the pregnancy, up until the last antenatal visit. | ATCM2_01 to ATCM2_26 | Antenatal record, free text | Self-reported at an antenatal visit in late pregnancy. Includes both prescription and over-the-counter medication. Free text is translated into ATC codes at the NBHW. Products that cannot be classified into the ATC system are categorized into groups such as 'HEMOFER' or 'OMEGA'. |

CS, Caesarean section. LMP, Last menstrual period. NBHW, National Board of Health and Welfare

^a^From the Register of the Total Population Register(1). For privacy concerns, except for Sweden/Nordic countries, researchers usually receive information of country of birth and citizenship according to continent.

^b^Birth weight for gestational age <2 standard deviations (SD) below (SGA) and >2 SD above (LGA) the mean, according to the Swedish sex-specific growth curve for normal fetal growth(2).

**Supplementary Table 2.** Onset of labour and mode of delivery. Swedish Medical Birth Register 2000-2020

|  | **Mode of delivery**^a^ | | |  |
| --- | --- | --- | --- | --- |
|  | Vaginal, non-instrumental | Vaginal, instrumental^b^ | Cesarean Section (CS) | Missing |
| **Onset of labour**^c^ |  |  |  |  |
| Spontaneous  Rate (%) | 81.5 | 7.0 | 7.2 | 4.4 |
| Induced  Rate (%) | 70.0 | 9.1 | 18.3 | 2.6 |
| CS before onset  Rate (%) | 0.1 | 0 | 96.1 | 3.8 |

^a^The mode of delivery groups are based on check-boxes in the obstetrical record, and refer to the last used methods during delivery. The three mode of delivery groups should be mutually exclusive.

Rates of invalid combinations are as follows: vaginal non-instrumental AND other (vaginal instrumental or CS): 0.04%; vaginal instrumental AND other (vaginal non-instrumental or CS): 0.48%; CS and others (vaginal non-instrumental or vaginal instrumental): 0.10%.

^b^Vaginal instrumental includes both vacuum extraction and forceps delivery (97.4% and 2.8%, respectively).

^c^The Onset of labour groups is based on check-boxes in the obstetrical record, and should be mutually exclusive. Rates of invalid combinations are as follows: spontaneous onset of labour AND other (induction or CS): 0.15%; induction of labour AND other 0.74%; CS before onset of labour AND other (spontaneous or induction): 0.38%.

Note: There are also variables created at the MBR for *any* use of vacuum extraction and forceps (SUGMARK and TANGMARK, respectively). These variables are based on checkboxes, maternal diagnostic and operation codes, and are created at the NBHW.

**Supplementary Table 3.** Distribution of birth weight for gestational age in the Swedish Medical Birth Register (MBR). Live singleton births 2000-2020 (n=2,203,837)

| Birth weight for gestational age^a^ (standard deviation scores) | No | **%** |
| --- | --- | --- |
| >5 | 1076 | 0,05 |
| >4 to 5 | 2279 | 0,10 |
| >3 to 4 | 13127 | 0,60 |
| >2 to 3 | 56597 | 2,57 |
| >1 to 2 | 304943 | 13,84 |
| -1 to 1 | 1497375 | 67,94 |
| -2 to <-1 | 277628 | 12,60 |
| -3 to <-2 | 40905 | 1,86 |
| -4 to <-3 | 4692 | 0,21 |
| -5 to <-4 | 555 | 0,03 |
| <-5 | 198 | 0,01 |
| Missing | 4462 | 0,20 |

^a^Gestational age is based on the variable GRBDS in the SMBR, which in turn is based on a hierarchy: estimated date of delivery by a) ultrasound (90.1%); b) last menstrual period (4.5%); and c) a clinical assessment (4.2%). Birth weight for gestational age is based on the ultrasonic-based reference curve for normal fetal growth(2).

**References**

1. Ludvigsson JF, Almqvist C, Bonamy AE, et al. Registers of the Swedish total population and their use in medical research. Eur J Epidemiol. 2016;31(2):125-36. doi:10.1007/s10654-016-0117-y

2. Marsal K, Persson PH, Larsen T, Lilja H, Selbing A, Sultan B. Intrauterine growth curves based on ultrasonically estimated foetal weights. Acta Paediatr. 1996;85(7):843-8.
