# Supplementary material for: The Swedish medical birth register during five decades: documentation of the content and quality of the register
Source: Eur J Epidemiol. 2023 Jan 3;38(1):109–20. doi: 10.1007/s10654-022-00947-5 (PMC9867659; doi:10.1007/s10654-022-00947-5)
Supplement: Supplementary file 2 — Supplementary Material 2 [file 10654_2022_947_MOESM2_ESM.doc]

Stockholm. Sweden

November 14, 2022

Dear Dr. Hofman,

We appreciate the comments from the peer reviewers and have revised the manuscript accordingly. We are happy to hear that it has now been accepted for publication.

The peer reviewers had only minor requests which we have commented on below.

**Reviewer #1:** Thanks to authors for the changes and clarifications.
One tiny editing detail: in the legend to Figure 3 the meaning of GRDBS was added, as requested.
However, the text now reads 'Best estimate of number of pregnancy in days, hierarchical variable'. I think it should read: 'Best estimate of number of pregnancy *duration* in days, hierarchical variable'
Even better would be: 'Best estimate of the duration of pregnancy in days, hierarchical variable'.
***Authors:***

1) *The legend of Figure 3 has been changed accordingly and GRDBS is now defined:*

*'Best estimate of the duration of pregnancy in days, hierarchical variable'*

2) *The GRDBS has been defined accordingly in the supplementary table 1.*

**Reviewer #2:** Thank you very much for the opportunity to review comment on your revised manuscript. My comments has been well addressed. Just one note regarding the validity. I am confident that the authors know the register and the validity of the varibles extremely well and that the direct computerized extraction of variables from medical records ensures similar data quality in MBR and medical records. However, a validation study between the gold standard (medical reports) and the register will confirm the fact quantitatively. That could be a future study. Unpublished data from Denmark show a general high validity of the register data but with decreasing validity with higher degree of details or less severe disorders/treatments/factors. This is based on thorough review of medical report in Denmark compared to register data.

***Authors:***

*We thank the peer reviewer for his/her comments. No changes have been made to the manuscript.*

Please note:

Two new documents (with the new Figure legend, and the suppl table 1) have been uploaded. All other documents remain unchanged.

Yours sincerely,

*Sven Cnattingius, Karin Källén, Anna Sandström, Henny Rydberg, Helena Månsson, Olof Stephansson, Thomas Frisell, and Jonas F Ludvigsson*
